# Molecular Basis for the Recognition of Herpes Simplex Virus Type 1 Infection by Human Natural Killer Cells

**DOI:** 10.3389/fimmu.2018.00183

**Published:** 2018-02-12

**Authors:** Hong-Sheng Dai, Michael A. Caligiuri

**Affiliations:** ^1^The Ohio State University Comprehensive Cancer Center, The James Cancer Hospital and Solove Research Institute, Columbus, OH, United States; ^2^Division of Hematology, Department of Internal Medicine, College of Medicine, The Ohio State University, Columbus, OH, United States

**Keywords:** natural killer cells, CD16, IgG, major histocompatibility complex I, NKG2D, herpes simplex virus type 1

## Abstract

Primary infection with Herpes simplex virus type 1 (HSV1) is subclinical or only mildly symptomatic in normal individuals, yet the reason for the body’s effective immune defense against this pathogen in the absence of antigen-specific immunity has not been well understood. It is clear that human natural killer (NK) cells recognize and kill HSV1-infected cells, and those individuals who either lack or have functionally impaired NK cells can suffer severe, recurrent, and sometimes fatal HSV1 infection. In this article, we review what is known about the recognition of HSV1 by NK cells, and describe a novel mechanism of innate immune surveillance against certain viral pathogens by NK cells called Fc-bridged cell-mediated cytotoxicity.

## Introduction

**Figure 1:**
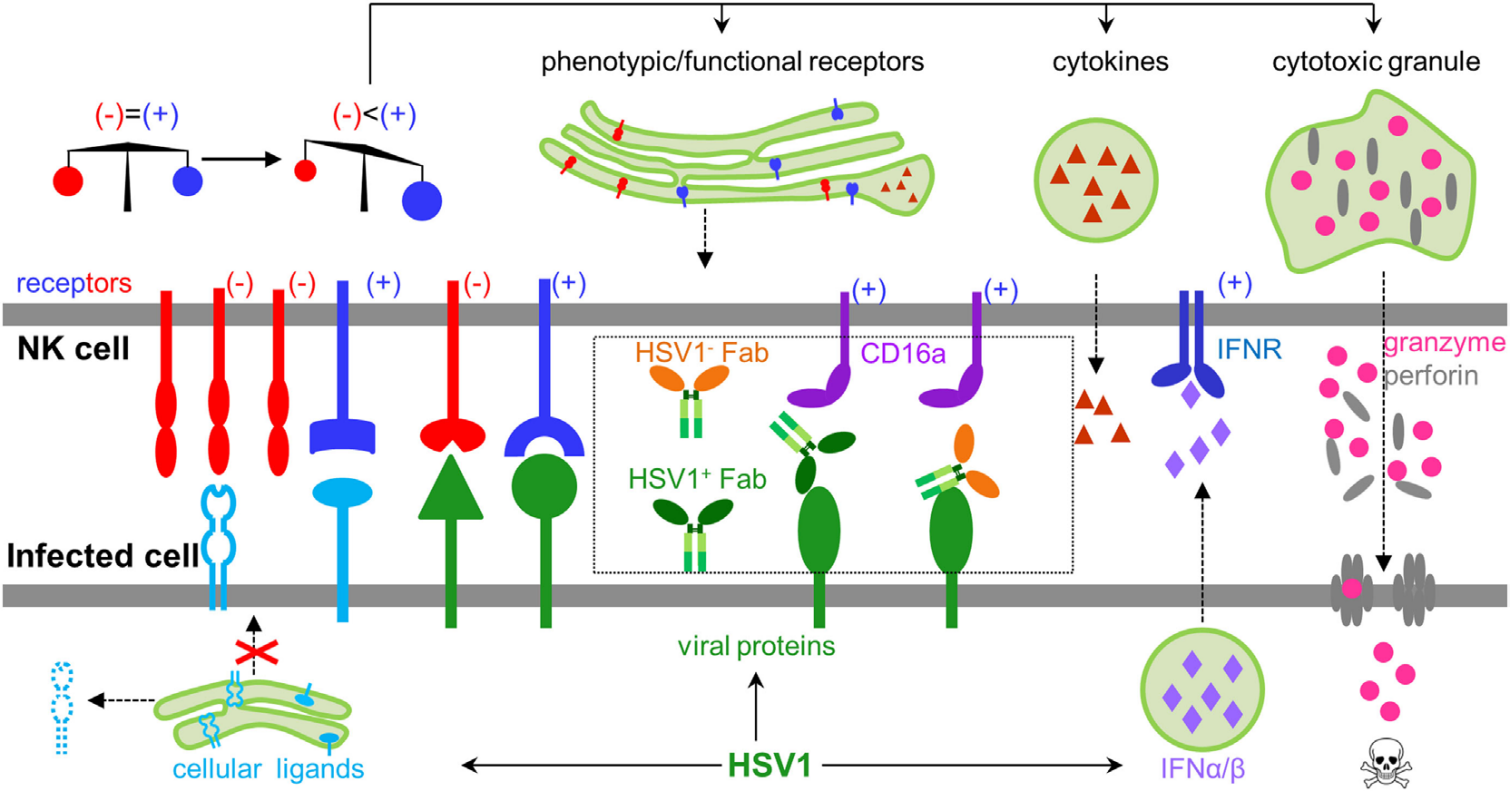
General rules regulating natural killer (NK) cell activity during herpes simplex virus type 1 (HSV1) infection. NK cells express both inhibitory and activating receptors, which balance activity of NK cells. HSV1 infection could shift the balance by secreting type I interferons, expressing activating ligands, decreasing inhibitory ligands, and engaging antibodies, which collectively lead to activating signals outweighing inhibitory signals. Activated NK cells produce cytokines, express functional proteins, and release granzyme and perforin to kill infected cells.

Natural killer (NK) cells are innate lymphoid cells capable of directly recognizing virally infected cells without prior antigen exposure (1), and constitute the first line of defense against herpes simplex virus type 1 (HSV1) infection (2). Patients with NK cell deficiencies can suffer severe, recurrent, and sometimes fatal HSV1 infection (3, 4). The functional status of NK cells is tightly regulated by signal inputs from a wide variety of NK cell activating and inhibitory receptors, which coordinately balance NK cell function to avoid autoimmune damage under normal physiology (1). HSV1 infection could diminish inhibitory signals and/or increase activating signals, leading to NK cell activation. HSV1 genomic DNA, viral RNA, and proteins are known to induce the production of type I interferons (5–7), which can greatly potentiate NK cell function during HSV1 infection (8). Upon activation, NK cells undergo dramatic phenotypic and functional changes, including expressing functional markers, secreting cytokines, releasing pre-stored perforin and granzyme B, and lysing target cells (Figure 1).

Natural killer cells express the low affinity Fcγ receptor FcγRIIIA/CD16a (CD16a hereafter), and are the major effector cells for antibody-dependent cell-mediated cytotoxicity (ADCC), which represents the main mechanism for NK cells to recognize and clear HSV1 infection after adaptive immunity is established. However, most primary HSV1 infections are asymptomatic or associated with only mild symptoms like fever and rash, suggesting HSV1 infection can be efficiently recognized and cleared by the innate immune response before an antigen-specific immune response is established (2).

Herpes simple virus type 1 is a member of the herpesviridae family and has a 150 kb double-stranded DNA genome, which contains 84 open reading frames and encodes 74 unique viral proteins (9). During lytic infection, HSV1 expresses a large amount of viral proteins in a kinetically regulated fashion, rendering the virus-infected cell susceptible to innate immune defense. During latency, expression of viral protein is minimized, thus hindering recognition of infected cells by the immune system. Although it has been suggested that some of these HSV1-encoded proteins might directly mediate recognition or evasion by immune cells (10–12), it remains controversial as to whether some viral or cellular proteins serve as cognate ligands for NK cells to sense HSV1 infection. In this review, we summarize the studies of molecules that are involved in the direct interactions between human NK cells and HSV1 lytic infection, discuss potential mechanisms for their action, and provide our interpretation for some conflicting studies.

## Contact Signals

Natural killer cell function is tightly regulated by an array of inhibitory and activating receptors that receive input by contacting cognate ligands present on target cells (13). Contact signals sparked by the collective interactions between NK cell receptors and target cell ligands are essential for the NK cell to release cytotoxic granules and kill target cells (13). Ligands for NK cell receptors mostly are cellular proteins (14), however, many viruses and fungi have been reported to express proteins that can directly bind NK cell receptors and modulate NK cells (15–18). Whether HSV1 expresses ligands that are directly recognized by NK cells has been controversial.

Heat- or UV-inactivated HSV1 viruses were shown to induce IFNα production and promote NK cytolysis, and it was suggested that viral proteins were directly responsible for this stimulatory immune responses (19–21). However, heat- or UV-inactivated HSV1 can still enter host cells and deliver viral genomic DNA, which in itself is a potent inducer of type I IFNs (22). Additional HSV1-encoded glycoproteins, including glycoprotein B (gB), gC, glycoprotein D (gD), and gH/gL, have been reported to activate NK cells (23–27), yet contradicting results have also been documented for each of these viral proteins (20, 21, 28). The discrepancy shown in these studies may partly arise from different experimental conditions: it nonetheless highlights the need for more well-designed studies to explore the potential role of viral proteins in directly regulating NK cell function.

Lytic HSV1 infection greatly changes the biosynthetic events of host cells, most prominently affecting host protein synthesis, traffic, and degradation (29). The pattern of cellular NK ligands expressed on target cells changes significantly following HSV1 infection, and provides a recognizable signal for NK cells to distinguish infected cells. Below, we discuss cellular ligands that change following HSV1 infection and HSV1 proteins that may contribute to direct NK cell recognition of HSV1.

### Major Histocompatibility Complex I (MHC I) Class I

In addition to presenting antigenic peptides to CD8+ cytotoxic T cells, the MHC I molecule is also the natural ligand for the inhibitory killer cell immunoglobulin-like receptors (KIRs) and the lectin-like inhibitory receptor CD94/NKG2a, both of which are expressed on human NK cells (1). Because of its ubiquitous presence on human tissue, MHC I molecules prevent NK cells from attacking healthy self, thereby preventing autoimmunity. Therefore, it has been hypothesized that downregulation of MHC I during viral infection through many different mechanisms could potentially release NK cells from self-inhibition and lead NK cells to recognize virally infected cells (30).

Fulfillment of antigen presentation by MHC I needs endogenous antigen peptides and the endoplasmic reticulum (ER) traffic protein: transporter associated with antigen processing (TAP). TAP pumps cytosol antigen peptides into the ER, where nascent MHC I molecules are loaded with antigen peptides and exported to the cell surface. Empty MHC I cannot pass the quality check and are not transported to the cell surface (31). HSV1 ICP47, encoded by the Us12 gene, is a soluble, cytosolic protein of 88 amino acid residues (32). ICP47 forms a long helical hairpin inserting into the central cavity that is formed by two TAP subunits (33). By plugging the TAP translocation channel, ICP47 precludes binding and traffic of antigenic peptide from the cytosol to the ER (33), and prevents transport of MHC I to the plasma membrane.

Several *in vitro* studies have confirmed that expression of ICP47 decreases surface MHC I on HSV1-infected human cells and consequently activates NK cells in co-culture (12, 34). However, ICP47 binds murine TAP1/2 poorly (30) and does not efficiently block traffic of mouse MHC I (35), making it difficult to test whether the downregulation of MHC I could affect NK cell activation and clearance of HSV1 infection *in vivo*. Both human cytomegalovirus (CMV) Us11 and mouse CMV (MCMV) m152 have been reported to decrease mouse MHC I presentation (32, 36, 37). Orr et al. thus constructed a recombinant HSV1 virus expressing HCMV Us11 or MCMV m152, and studied the effect of MHC I downregulation on the immune recognition of HSV1 infection in mice (34). The recombinant HSV1 viruses nonetheless did not decrease MHC I expression on mouse cell lines more than the wild type HSV1 (34). Thus, importance of downregulating MHC I for clearance of HSV1 infection by NK cells *in vivo* remains unresolved and awaits better models to resolve this issue.

### NKG2D Ligands

NKG2D is one of the major NK cell receptors involved in recognition and killing of tumor cells and virus-infected cells (38). In humans, NKG2D is engaged by several ligands, namely MHC class I polypeptide-related sequence A and B (MICA and MICB) and the UL16-binding proteins 1–6 (ULBP1–6) (39). It has been reported that an HSV1-infected cell line had lower expression of MICA and ULBP2, which could potentially help HSV1-infected cells to evade recognition by NK cells (40, 41). Although the exact mechanism for this downregulation of MICA and ULBP2 is unknown, the recycling of membrane protein and general inhibition of *de novo* synthesis of cellular proteins during HSV1 infection might contribute to the decrease of NKG2D ligand expression (29). NK cells from patients with active HSV1 infection had a higher level of NKG2D (40), possibly induced by an elevated level of type I IFN during HSV1 infection (42). The increased NKG2D levels may sensitize NK cells and counteract the effect of decreased NKG2D ligand expression on HSV1-infected cells.

### Glycoprotein D

Pierre Lebon reported that diploid cells infected with HSV1 can induce IFNα production by peripheral blood mononuclear cells, and that HSV1 gD was responsible for this biological effect (23). HSV1 gD, encoded by the Us6 gene, is the major glycoprotein mediating entry of HSV1 into host cells. It binds two cellular receptors: herpesvirus entry mediator (HVEM) and nectin1 (43). While nectin1 has not been identified to have any regulatory function, HVEM is a member of the tumor necrosis factor alpha superfamily and plays very diverse roles in modulating T-cell function by activating both inflammatory and inhibitory signaling pathways (44).

Herpesvirus entry mediator binds many functionally diverse cellular proteins, including LIGHT (lymphotoxin-like, exhibits inducible expression, and competes with herpes simplex virus glycoprotein D for HVEM, a receptor expressed by T lymphocytes), lymphotoxin-α, B and T lymphocyte attenuator (BTLA), and CD160. Crystal structure of the HVEM-ligand complex shows that the binding sites on HVEM for gD, BTLA, and CD160 are overlapping or very close (45). HVEM is ubiquitously expressed by both human and mouse immune cells (our unpublished data). A recent study showed that HVEM was required for IFNα production following *Listeria* infection in mice (46). Collectively, these results suggest that HVEM might not only be the entry mediator, but also the immune sensor for HSV1 infection. However, we recently reported that expression of gD makes glioma resistant to NK cell cytotoxicity (47), and others reported that blocking gD did not affect the response of NK cells to HSV1-infected cells (20, 27, 28). Thus, the role of gD in NK cell response to HSV1 infection is yet to be clarified, similar to the role of HVEM in this process.

### Glycoprotein B

Herpes simplex virus type 1 gB promotes viral attachment through interaction with cell surface heparin sulfate (48), and also plays an essential role in mediating membrane fusion (49). HSV1 gB has been reported as having a role in the NK cell lysis of HSV1-infected endothelial cells (24–26). A lower lysis of target cells infected with HSV1 was observed when viruses were deficient in gB, or when Fab fragments of a gB-specific antibody were added to block gB (24–26). Leoni et al. reported that gB was able to physically interact with toll-like receptor-2 (TLR2) (27). In another study, Kim et al. reported that the activation of NK cells by UV-inactivated HSV1 virions was directly mediated by TLR2 (20). They showed that UV-inactivated HSV1 virions could bind the endothelial cell line HEK when ectopically expressing TLR2, but not native HEK2 cells that lack TLR2. However, the authors did not confirm the expression of TLR2 on NK cells, or whether the activation of NK cells by HSV1 was mediated by the TLR2-gB interaction (20). The expression of TLR2 in NK cells is still controversial. Although TLR2 mRNA has been detectable in human NK cells, TLR2 protein has only been noted on decidual NK cells (50), but not on the surface of human circulating NK cells (51–55). Another study also showed TLR2 was not required for recognizing HSV1 glycoproteins (28). Experiments using different strains of HSV1 may have contributed to the discrepancies seen within these studies. Collectively, these data make it difficult to draw a conclusion regarding the role of gB in mediating NK cell recognition of HSV1-infected cells.

### Other Cellular Ligands and Viral Proteins

Fitzgerald-Bocarsly et al. reported that expression of HSV1 immediate early genes caused the increased susceptibility of HSV1-infected fibroblast cells to NK cell lysis (56). Chisholm et al. further pinpointed this NK cell stimulating function to ICP0 (11). ICP0 is cytosolic protein and theoretically should not be able to activate the NK cell directly. The investigators found ICP0 did not change the expression of MHC I or of NKG2D ligands, but induced the expression of some unidentified ligands for natural cytotoxicity receptors NKp30, NKp44, and NKp46 (11). However, during HSV1 infection, cellular proteins are only rarely unregulated due to wide spread disruption of host mRNA (29). The finding that ICP0 expression induces expression of ligands for natural cytotoxicity receptors, if confirmed, would be very helpful for identifying these cellular ligands important for the function of NK cells.

## The Role of IgG

**Figure 2:**
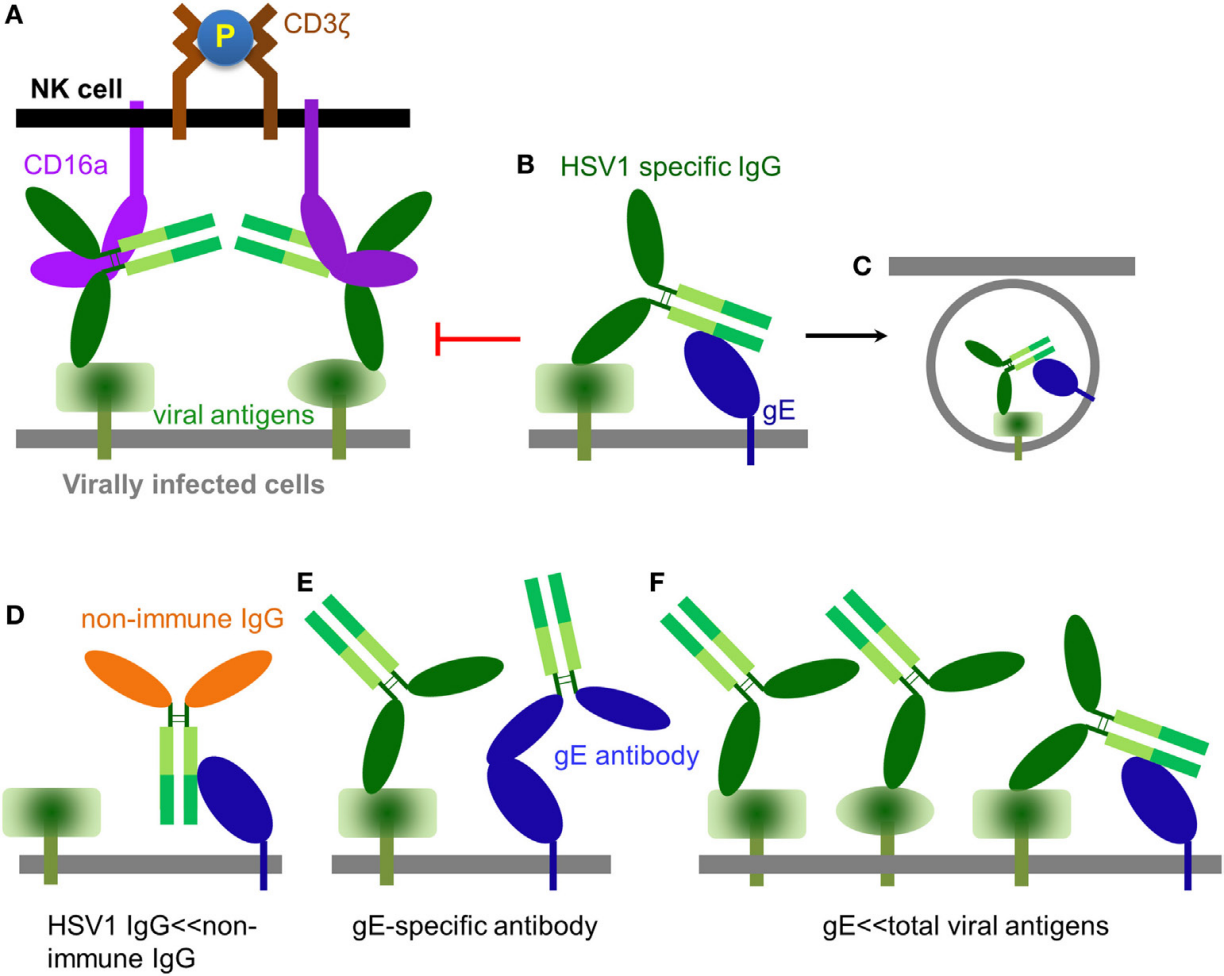
The role of IgG in modulating natural killer (NK) cell function. **(A)** An HSV1-specific antibody binds to a specific viral antigen and activates CD16a(+) NK cells through classical antibody-dependent cell-mediated cytotoxicity (ADCC). **(B)** It has been proposed that antibodies directed against herpes simplex virus type 1 (HSV1) antigens could from a bipolar bridge between a specific viral antigen and the HSV1 IgGFc-binding glycoprotein glycoprotein E (gE), thereby preventing ADCC (64). **(C)** Endocytosis of viral antigens mediated by the hypothetical antibody bipolar bridge. **(D)** During primary HSV1 infection, non-immune IgG dominates while a primary immune response is being generated. In this setting, non-immune IgG can directly bind gE *via* its interface with the CH2–CH3 region of IgG. **(E)** gE-specific antibody can bind gE *via* its IgG Fab and prevent gE from binding another IgG at its CH2–CH3 region. **(F)** HSV1 infection produces large amounts of viral antigens of which gE accounts for only a small fraction. Thus, only a small fraction of the total HSV1-specific IgG can potentially form the bipolar bridge on the HSV1-infected cell.

Globally, 70% of the human population is estimated to be HSV1 seropositive (57). Once adaptive immunity against HSV1 is generated, it is believed that HSV1-specific antibodies could exist in the serum at high titer throughout human life and effectively prevent infection (58), which is consistent with the rarity of recurrent HSV1 infection in immune competent populations even though repetitive HSV1 exposure occurs often. NK cells express CD16a and are the major effector cells of ADCC (59). CD16a is a type I transmembrane protein, whose extracellular domain binds Fcγ at the hinge region (60) and whose transmembrane helix and intracellular domain couple with the signal transducer CD3ζ (61, 62). NK cells utilize CD16a to recognize antibody-bound pathogens, including infected cells. Binding of immune complex consequently clusters and phosphorylates CD3ζ and eventually leads to the activation of the NK cell and lysis of infected cells (Figure 2A) (63).

### Antibody Bipolar Bridging

Herpes simplex virus type 1 expresses an IgGFc-binding protein glycoprotein E (gE), which binds human IgG1, IgG2, IgG4, but not IgG3. HSV1 gE alone binds Fc of IgG with low affinity, however, it can form a heterodimer with glycoprotein I (gI) (65). Although gI has no direct contact with IgG, the gE–gI complex binds Fc with much higher affinity than gE alone (66, 67). HSV1 gE–gI complexes have been shown to participate in “antibody bipolar bridging,” whereby a single anti-HSV1-specific IgG antibody simultaneously binds to an HSV1 antigen using its Fab region and to gE *via* its Fc region (Figure 2B) (64). It has been proposed that such antibody “bipolar bridging” could block the access of the NK cell’s CD16a to the Fc portion of the anti-HSV1-specific IgG antibody (68, 69), and induce endocytosis of viral antigens (Figure 2C) (70). Therefore, antibody bipolar bridging was proposed to reduce classical ADCC and provide a mechanism for innate immune evasion following HSV1 infection (68, 69).

**Figure 3:**
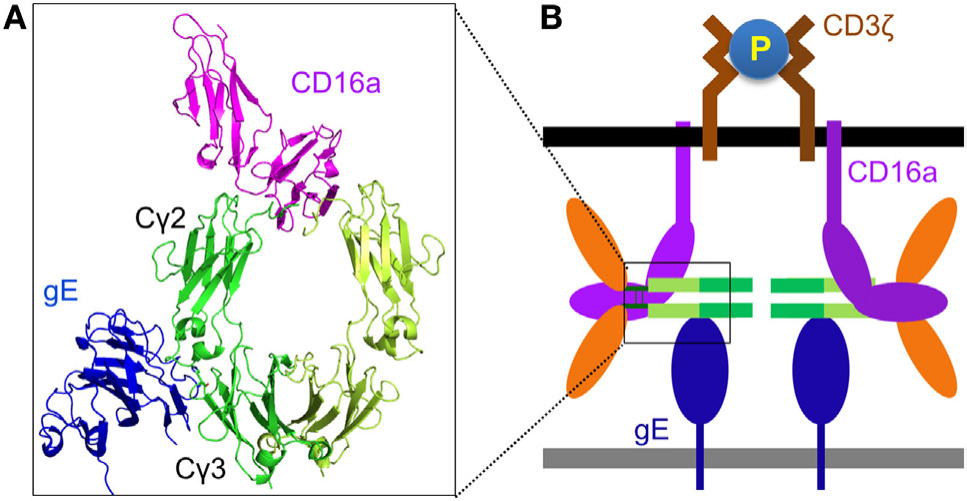
Structural basis for Fc-bridged cell-mediated cytotoxicity (FcBCC). **(A)** Model structure of gE-IgG1Fc-CD16a ternary complex showing the non-overlaping binding of herpes simplex virus type 1 (HSV1) glycoprotein E (gE) and CD16a to IgGFc. CD16a is shown as magenta, gE is shown as blue, two monomers of IgGFc dimer are shown as green and lime. **(B)** A new type of IgG-mediated natural killer (NK) cell activation called FcBCC is shown. IgGFc molecules bound by gE are still accessible for CD16a and able to cluster CD3ζ, thereby activating NK cells to kill HSV1-infected targets prior to the development of a antibody-specific immune response.

Although the antibody bipolar bridge has been tested in several experiments (68, 69), its existence and role in inhibiting ADCC *in vivo* remains controversial for several reasons. First, HSV1-specific antibody only accounts for a small fraction of the whole human IgG pool (20). The probability of gE to bind a predominant non-HSV1-specific IgG molecule is much greater than the probability of gE interacting with an HSV1-specific antibody that is already bound on the same infected cell, even without considering the steric hindrance that might not favor the forming of such bipolar bridge (Figure 2D). Second, gE is a major HSV1 antigen and gE-specific antibodies exist in most HSV1 seropositive serum. The gE-specific antibodies use Fab to bind gE and potentially block gE interaction with the IgGFc of other antibodies (Figure 2E). Third, gE constitutes only a small portion of all viral antigens that express HSV1-infected cells and HSV1-specific antibodies coated on HSV1-infected cells far outnumber all that gE could bridge (Figure 2F). Therefore, the bipolar bridge of IgG and gE, even if it existed, should not contribute significantly enough to reduce ADCC of HSV1-infected cells in a seropositive individual. Lastly and most importantly, the crystal structure of the gE-IgG1Fc complex shows that gE binds IgG1Fc at the CH2–CH3 interface, a site that is distinct from the Fcγ hinge region where CD16a binds (71). Therefore, HSV1 gE and CD16a are not mutually excluded from binding the same IgG, and the assumption that gE could prevent CD16a from binding the same IgG molecule is without structural basis (Figures 3A,B).

### Fc-Bridged Cell-mediated Cytotoxicity (FcBCC)

Instead of inhibiting NK cell cytotoxicity, we recently reported that the expression of the Fc-binding protein gE on HSV1-infected glioma cells actually stimulated NK cell activation and cytotoxicity, and co-expression of gE and gI further enhanced NK cell activation. Primary human NK cells are naturally coated with IgG molecules, and we found that the response of human NK cells toward gE or gE–gI directly correlated with the individual’s NK cell surface density of IgG (47). Further, as noted earlier, the crystal structure of the gE-IgG1Fc complex showed that gE binds IgG1Fc at its CH2–CH3 region, a site that is distinct from the Fcγ hinge region, where CD16a binds (71). We, therefore, proposed that HSV1 gE, IgGFc, and CD16a could form a ternary complex (Figures 3A,B). The gE-IgGFc-CD16a complex was confirmed and responsible for relaying the activating signal for NK cells upon encounter with HSV1-infected glioma cells in the absence of specific anti-HSV1 antibodies (47).

Although HSV1 gE does not bind mouse IgG (72), mouse NK cell FcγR (CD16a) binds human IgG with high affinity (36). This led us to test whether human IgGFc alone could bridge mouse NK cells and HSV1-infected cells, and promote clearance of HSV1 infection *in vivo*. We found infusion of human IgG1Fc fragments alone protected mice from lethal HSV1 infection in a manner dependent on NK cells and gE, as did other human IgG1 therapeutic antibodies not targeting any HSV1 antigens (47). It is well established that protective functions of IgG against infection and cancer require utilization of both its Fab and Fc domains. NK cell activation *via* gE-IgGFc-CD16a differs from the classical IgG function of ADCC by not requiring any antigen-specific antibody, and limits virus infection before the establishment of adaptive immunity. We thus named this process of innate immune recognition FcBCC (Figure 3B).

Fc-bridged cell-mediated cytotoxicity represents a previously unappreciated mechanism of innate immune cell recognition of and response to a primary viral infection mediated only by the Fc domain of IgG bound to FcγR and recognizing the pathogen expressing an Fc binding protein, as well as naked CD16a recognizing the Fc domain of IgG bound to the infected cell’s Fc-binding protein. The experimental evidence for FcBCC is consistent with the observation that most primary HSV1 infections are clinically asymptomatic and/or self-limited. It is also highly likely that FcBCC is responsible for the rapid NK cell clearance of oncolytic HSV1 in the setting of malignant glioma (73), and clearance of infection by many other members of the herpesviridae family encoding similar or identical Fc-binding proteins (37, 69). FcBCC of HSV1-infected glioma by CD16a(+) NK cells is abrogated in the absence of the HSV1 binding protein gE (47). Previous studies suggesting that NK cell activation is enhanced in the absence of HSV1 gE were all conducted in the presence of anti-HSV1 antibodies (64, 68, 69); the caveats of the conclusions drawn from those studies were discussed above and illustrated in Figures 2D–F. It is possible that the anti-HSV1 antibodies induce endocytosis of viral antigens in a gE–gI dependent fashion (70), thereby reducing NK cell activation *via* classical ADCC. Under this circumstance, the absence of HSV1 gE would inhibit the endocytic process, resulting in more surface expression of viral targets and consequently improved classical ADCC.

## Conclusion and Future Directions

Natural killer cell recognition of HSV1 infection is the synergistic result of multilayer activating and inhibiting signals, involving soluble factors, contact signals, and IgG molecules. IgG plays the central role for recognition and clearance of HSV1 infection by NK cells during both primary and recurrent infection. During primary infection, non-immune IgG can coat infected cells *via* the interaction of IgGFc with gE and facilitate clearance of HSV1 infection by CD16a(+) NK cells through FcBCC. Once adaptive immunity is established, HSV1 infection is recognized and bound by HSV1-specific IgG and cleared by NK cells through classical ADCC.

It is relatively less studied how other contact signals contribute to the NK cell activation. It is technically challenging to dissect the contribution of individual viral components to evade or activate NK cell recognition, because (1) these viral components may only exert the effect in special host cells, at particular stages of infection, and dependent on the strain of HSV1 virus; (2) they share redundancies in shaping the function of NK cells; thus removing one would not be enough to show the difference in affecting NK cell function; (3) multi-functional nature of many viral proteins makes it difficult to compare data acquired from wild type HSV1 viruses with those from gene-specific deficient HSV1 viruses, because loss of viral genes may change not only the phenotype related to interactions with NK cells, but may also impact the virus’ replication and life cycle. HSV1 is a human pathogen and patients with NK cell deficiency almost always develop severe HSV1 infections. So, a better way to study the interaction of HSV1 infection and NK cells is to look into the molecular basis of the deficiency causing the patient susceptibility for HSV1 infection. This will help expand our knowledge about NK cells beyond HSV1 infection.

## Author Contributions

HSD and MAC jointly wrote the paper and drew the figures.

## Conflict of Interest Statement

A provisional patent for Fc-bridged cellular-mediated cytotoxicity has been filled by the authors with the US patent office: U.S. Application No. 62/452,111_T2017-083. The reviewer FC and handling editor declared their shared affiliation.
